# Petal‐specific RNAi‐mediated silencing of the phytoene synthase gene reduces xanthophyll levels to generate new *Oncidium* orchid varieties with white‐colour blooms

**DOI:** 10.1111/pbi.13179

**Published:** 2019-08-19

**Authors:** Yao‐Chung Liu, Chao‐Wei Yeh, Jeng‐Der Chung, Chih‐Yu Tsai, Chung‐Yi Chiou, Kai‐Wun Yeh

**Affiliations:** ^1^ Institute of Plant Biology National Taiwan University Taipei Taiwan; ^2^ Taiwan Forestry Research Institute Council of Agriculture Taipei Taiwan; ^3^ Climate Change and Sustainable Development Research Center National Taiwan University Taipei Taiwan; ^4^ Present address: Department of Post‐Modern Agriculture College of Applied Sciences MingDao University Changhua 523 Taiwan

**Keywords:** Oncidium, RNA‐mediated silencing, phytoene synthase, petal‐specific promoter, transgenic orchid

Colour is a naturally important characteristic in plant biology. It is also an important trait for fruits and vegetables. Three major groups of pigments—betalains, flavonoids and carotenoids—are responsible for pigmentation in plants. Carotenoids, the most widely distributed pigment, display diverse colours, ranging from yellow and orange to deep red. The first step of carotenogenesis is the dimerization of two molecules of geranylgeranyl pyrophosphate (GGPP) to phytoene by the enzyme phytoene synthase (PSY). This is the rate‐limiting step in the carotenoid biosynthesis route and has long been considered a ‘bottleneck’ in the pathway. Previous plant biotechnology studies have enhanced the carotenoid levels to increase the nutritional value of crops and fruits by conventional breeding and genetic engineering. In the past two decades, on the purpose to alleviate the public health problem of vitamin A deficiency (VAD) in large number of countries, the *Crt B* (for *phytoene synthase*)*, Crt I* (for *phytoene desaturase*) and *Crt Y* genes (for *lycopene ß‐cyclase*) of bacteria were singly or in combination overexpressed in various crops to generate provitamin A‐rich foods. For example, the Golden rice (Ye *et al*., 2000), Brassica golden seed (Shewmaker *et al*., 1999), Golden potato tuber (Diretto *et al*., 2010), maize of high carotenoid endosperm (Aluru *et al*., 2008) and tomato (Fraser *et al*., 2002) were successfully enhanced with the predominant accumulation of ß‐carotene. In addition, the RNAi‐mediated technology to down‐regulate *DET 1* (*De‐etiolated 1* gene, a negative regulator of light signal transduction in tomato) expression and to obtain the increasing carotenoid level in tomato has also been carried out (Davuluri *et al*., 2005). With regard to the flower colour alteration using genetic engineering, considerable progress has been made in the past 20 years. However, those works were concentrated on flavonoid modification, such as blue rose, carnation and petunia (Tanaka *et al*., 2010). Thus far, the carotenoid modification to change flower colour was only reported on transgenic chrysanthemum, by knockdown of *CCD4* to generate vivid yellow flower from white flower (Ohmiya, 2009). Next to the success of transgenic chrysanthemum, silencing of *phytoene synthase* by RNAi to reduce carotenoid content in *Oncidium* floral tissues is performed and achieved in this paper.

**Figure 1:**
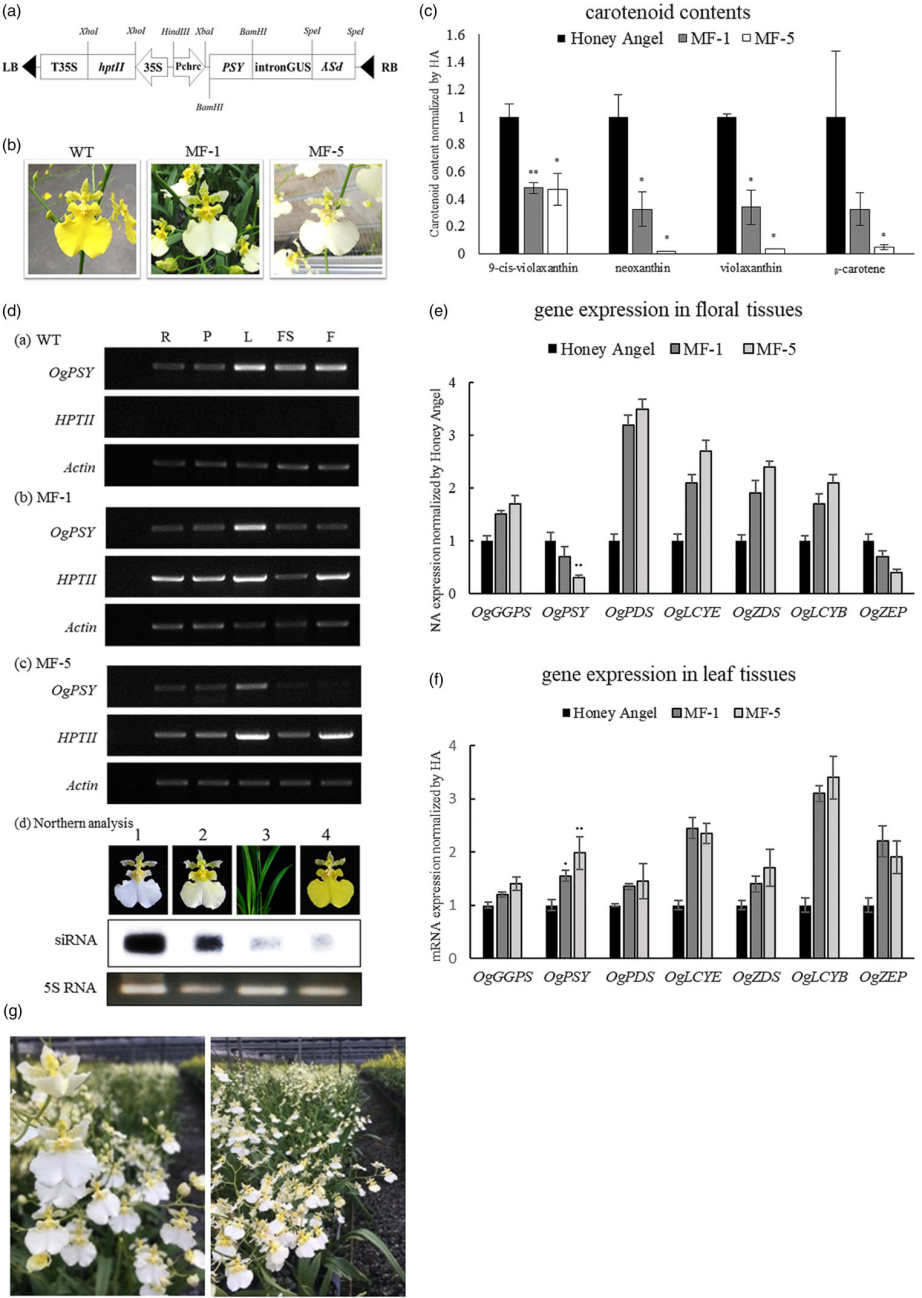
RNAi construct transformation, generation of white transgenic *Oncidium* varieties, and characterization of carotenoid compounds and relevant gene expression. (a) Schematic representation of hpRNA silencing vectors. Two 150‐bp *PSY* ORF fragments were inversely fused to both ends of the spacer DNA fragment, GUS intron (750 bp), and driven by Pchrc promoter. (b) Photographs of the transgenic *Oncidium* orchids harbouring PSY‐RNAi constructs. From left to right: WT orchid (yellow), transgenic orchid MF‐1 (yellowish white); and transgenic orchid MF‐5 (white). (c) Carotenoid levels in the floral tissues of the three *Oncidium* orchids. (*significantly different compared to control at *P* < 0.05, **significantly different compared to control at *P* < 0.01, by t tests, *n* = 2) (d) Analysis of *OgPSY* RNA expression in transgenic orchids. Semi‐quantitative real‐time PCR analysis of *OgPSY* expression in WT orchid; transgenic orchid MF‐1; and transgenic orchid MF‐5. R, root; P, pseudobulb; L, leaf; FS, flower stem; F, floral tissues; *HPTII, hygromycin phosphotransferase II* gene. Northern analysis of siRNA for the floral tissues of MF‐5 (lane 1), MF‐1 (lane 2), leaves of MF‐5 (lane 3) and the WT orchid (lane 4). The α‐^32^P‐labelled 18‐bp RNA probe, 5′‐GUUAGAUACACACCACGU‐3′, was used to hybridize. (e) and (f) Real‐time quantitative PCR analysis of the expression patterns of genes involved in carotenoid biosynthesis in floral tissues and in leaf tissues. (*significantly different compared to control at *P* < 0.05, **significantly different compared to control at *P* < 0.01, by t tests, *n* = 3) (g) The white transgenic orchid plants (MF‐5) grown in a greenhouse. They displayed the stable traits.

*Oncidium* orchids are native to Middle and South America. *Oncidium* hybrids are the second most popular varieties of orchids in the orchid industry, next to the *Phalaenopsis* spp. The past breeding efforts have produced many commercial cultivars such as *Oncidium* Sharry Baby, Sweet Sugar, Gower Ramsey and Honey Angel, which are widely available in the current global market. Among these cultivars, *Onc*. Gower Ramsey and *Onc*. Honey Angel have been the most popular cultivars in the commercial cut‐flower market in Asian countries. The current *Onc*. Gower Ramsey and Honey Angel have the drawbacks of reproductive sterility, which makes conventional breeding difficult to generate new variety. Although several somatic mutants thus far have been occurred in tissue‐culture propagation process (Chiou *et al*., 2010), their floral characters were difficult to meet customer favourites, for example, the two somaclonal mutant varieties—White Jade and Sunkist. The former was caused by the natural mutation of methylation effect on *OgCCD1* (C*arotenoid* Cleavage *Dioxygenase* 1, catabolizing carotenoid metabolites) promoter. The elevated expression level of *OgCCD1* resulted in white floral colour, but with negative side effect of tiny, sparse florets and short vast life; the latter cause by the down‐regulation of *OgHYB* and *OgZEP* in carotenoid biosynthetic pathway exhibited the un‐delightful dark brownish florets (β‐carotene accumulation). Efforts were taken to overexpress *OgCCD1* gene to obtain white‐colour blooms with good phenotype characters and the results were negative (data not shown). Therefore, an attempt to obtain a white‐flowered *Oncidium* variety was designed by blocking the carotenoid biosynthesis pathway in the yellow‐coloured flowers of an *Oncidium* hybrid, such as Honey Angel. Herewith, we constructed a chimeric hairpin‐RNA (hpRNA) transfer‐DNA (T‐DNA) vector targeting *PSY* mRNA (Figure 1a). After blast analysis on the five *PSY* nucleotide sequences from other four monocot plant species (rice, wheat, maize, *Phalaenopsis*) in NCBI data bank, the 150‐bp cDNA region (+51~ +200 bp in ORF) of the *OgPSY* gene (accession number: FJ859989; refer to Chiou *et al*., 2010), containing a 22‐bp conserved sequence of 100% homology across corresponding complementary DNA (cDNA) sequences, was chosen for RNAi targeting. P*chrc*, a previously identified promoter region (1.5 kb) of the *CHRC* gene with specific expression in floral tissues (Chiou *et al*., 2008), was employed to drive the DNA segment of chimeric hairpin‐RNA. The hpRNA T‐DNA vector was delivered into protocorm‐like body (PLB) of WT (*Onc*. Honey Angel) by *Agrobacterium*‐mediated transformation. The transformed PLB cultures, exhibiting an approximately 20% transformation efficiency, were screened under hygromycin (30 ppm), regenerated to form seedlings and then planted in pots for two years. After flowering, 50 transgenic orchids harbouring the *PSY*‐RNAi construct were obtained. These transgenic orchids—approximately 60% of which showed the RNAi effect—displayed whitish to white florets. Subsequently, two independent lines were selected for further study. One displayed a yellowish‐white bloom designated as ‘MF‐1’, and the other showed white blooms were designated as ‘MF‐5’ (Figure 1b). Unlike the previous work employing CaMV 35S promoter, it caused the transgenic *Oncidium* orchids growth‐arrested and non‐flowering (Liu *et al*., 2014). The current two transgenic lines were very similar to WT orchids in terms of growth rate and phenotypic traits, such as floral size, floral stem, floret number and leaf morphology. Comparing to the former somaclonal mutant variety, White Jade, which displayed tiny, few florets in floral stem and early wilting (Chiou *et al*., 2010), it suggests that RNAi technology is an effective and under‐controllable genetic manipulation. Upon analysis of carotenoid levels in floral tissues, MF‐1 and MF‐5 were found to contain 40% of 9‐cis‐violaxanthin levels relative to WT orchid. Further, while MF‐1 contained 30% of neoxanthin, violaxanthin and β‐carotene levels relative to the recipient, MF‐5 contained only 1–3% of these compounds (Figure 1c).

As shown in Figure 1d, WT orchid showed high *OgPSY* (designation given to the *PSY* gene of *Oncidium* orchids) expression levels in floral tissues, leaves and flower stems; in comparison, the *OgPSY* expression levels in MF‐1 were lower, and those in MF‐5 were the least among them (Figure 1d). Meanwhile, *HPTII* was constitutively expressed in MF‐1 and MF‐5, while no expression was observed in WT orchid (Figure 1d). Moreover, northern blot analysis, performed to estimate the extent of *OgPSY* silencing in the floral tissues of both transgenic orchid lines, demonstrated the presence of *OgPSY*‐derived small interfering RNAs (siRNAs) in the floral tissues of MF‐1 and MF‐5 (Figure 1d—northern analysis). It showed more extensive disruption of *OgPSY* expression in MF‐5 than in MF‐1. Upon comparing the *OgPSY*‐derived siRNA levels in the floral and leaf tissues of MF‐5 (Figure 1d—northern analysis, lane 1& 3), although there seemed little siRNA levels existing in the leaves and WT, it could demonstrate that the P*chrc* promoter was activated specifically in petal tissues. The data clearly indicated the successful silencing of *PSY* expression specifically in the floral tissues of the white orchid varieties. We also analysed the mRNA levels of the carotenoid biosynthesis pathway genes in floral tissues by Q‐PCR. The data demonstrated that the carotenoid pathway genes exhibited enhanced expression level, except for *OgPSY*, which was down‐regulated to 70% in MF‐1 and 20% in MF‐5 (Figure 1e). This enhanced expression can be explained as a compensation effect for the flux in the entire carotenoid pathway. Upon analysing the expression patterns in leaf tissues, it was interesting to note that all carotenoid pathway genes in the transgenic orchids exhibited higher expression levels than in the WT (Figure 1f). Although the gene silencing was not targeted to the leaf, the side effects were present.

The phenotypic stability of a product is a priority for commercialization purposes. Because the *Oncidium* hybrid has lost pollen fertility for sexual reproduction, asexual multiplication by tissue culture is the sole way for propagating its seedlings. In this study, we multiplied the MF‐1 and MF‐5 plants from the P_0_ stage by culturing tiny meristematic bud tissues on half‐strength Murashige & Skoog (MS) medium containing 30 ppm hygromycin. Through PLB and shooting steps, we were able to produce two thousand P_1_ seedlings of each individual line after 2 years. Seedlings were grown at a temperature of 30°C/25°C (day/night), and the light intensity was controlled at the PPFD (photosynthetic photon flux density) of approximately 300 μmol/m^2^/s. Bloom colour was used as the main parameter for assessing phenotypic stability. Throughout the 2‐year cultivation period, the P_1_ plants displayed stable white pigments in their flowers, approximately 85% and 88% in MF‐1 and MF‐5, respectively. Further, we selected the best one from the P_1_ population and propagated them by tissue culture again. We then evaluated the phenotypic traits of the P_2_ population over another 2‐year period. As shown in Figure 1g, the P_2_ plants of both lines produced approximately 100% whitish/white florets, suggesting that the RNAi‐mediated suppression of *OgPSY* expression was stably inherited in this population through consecutive selection. The results of analysis of *OgPSY* expression revealed that the *OgPSY* mRNA levels in the P_2_ plants were as low as those in the P_0_ transgenic orchids and lower than those in WT (data not shown). These results strongly confirmed the genetic stability of the RNAi‐mediated transformation. In conclusion, our work demonstrates that down‐regulation of *PSY* expression by RNAi technology under the regulation of a tissue‐specific promoter, Pchrc, is effective to modify the flower colour specifically in floral tissues.
